# *Lactobacillus* spp. act in synergy to attenuate splenomegaly and lymphadenopathy in lupus-prone MRL/*lpr* mice

**DOI:** 10.3389/fimmu.2022.923754

**Published:** 2022-07-28

**Authors:** Xavier Cabana-Puig, Qinghui Mu, Ran Lu, Brianna Swartwout, Leila Abdelhamid, Jing Zhu, Meeta Prakash, Thomas E. Cecere, Zhuang Wang, Sabrina Callaway, Sha Sun, Christopher M. Reilly, S. Ansar Ahmed, Xin M. Luo

**Affiliations:** ^1^ Department of Biomedical Sciences and Pathobiology, College of Veterinary Medicine, Virginia Tech, Blacksburg, VA, United States; ^2^ Graduate Program in Translational Biology, Medicine, and Health, Virginia Tech, Roanoke, VA, United States; ^3^ Carilion School of Medicine, Virginia Tech, Roanoke, VA, United States; ^4^ Department of Development and Cell Biology, University of California, Irvine, Irvine, CA, United States; ^5^ Edward Via College of Osteopathic Medicine, Blacksburg, VA, United States

**Keywords:** lupus, gut microbiota, *Lactobacillus*, type 1 regulatory T cells, memory T cells, double-negative T cells

## Abstract

Commensal bacteria and the immune system have a close and strong relationship that maintains a balance to control inflammation. Alterations of the microbiota, known as dysbiosis, can direct reactivity to self-antigens not only in the intestinal mucosa but also at the systemic level. Our laboratory previously reported gut dysbiosis, particularly lower abundance of bacteria in the family *Lactobacillaceae*, in lupus-prone MRL/*lpr* mice, a model of systemic autoimmunity. Restoring the microbiota with a mix of 5 different *Lactobacillus* species (spp.), *L. reuteri, L. oris, L. johnsonii, L. gasseri* and *L. rhamnosus*, attenuated lupus-liked clinical signs, including splenomegaly and lymphadenopathy. However, our understanding of the mechanism was limited. In this study, we first investigated the effects of individual species. Surprisingly, none of the species individually recapitulated the benefits of the mix. Instead, *Lactobacillus* spp. acted synergistically to attenuate splenomegaly and renal lymphadenopathy through secreted factors and a CX_3_CR1-dependent mechanism. Interestingly, oral administration of MRS broth exerted the same benefits likely through increasing the relative abundance of endogenous *Lactobacillus* spp. Mechanistically, we found increased percentages of FOXP3-negative type 1 regulatory T cells with administration of the mix in both spleen and mesenteric lymph nodes. In addition, oral gavage of *Lactobacillus* spp. decreased the percentage of central memory T cells while increasing that of effector memory T cells in the lymphoid organs. Furthermore, a decreased percentage of double negative T cells was observed in the spleen with the mix. These results suggest that *Lactobacillus* spp. might act on T cells to attenuate splenomegaly and lymphadenopathy. Together, this study advances our understanding of how *Lactobacillus* spp. attenuate lupus in MRL/*lpr* mice. The synergistic action of these bacteria suggests that multiple probiotic bacteria in combination may dampen systemic autoimmunity and benefit lupus patients.

## Introduction

Recently generated evidence suggested that gut microbiota and the immune system interact to maintain tissue homeostasis (1–5), but it is unclear how disturbances in this interaction contribute to the pathogenesis of systemic lupus erythematosus (SLE). Our research team was the first to describe changes of gut microbiota in lupus-prone compared to control mice. Specifically, MRL/*lpr* lupus-prone mice have a decrease in *Lactobacillaceae* and an increase of *Lachnospiraceae* species (6), and administration of a mixture of 5 strains of *Lactobacillus* spp. into lupus-prone MRL/*lpr* mice *via* oral gavage corrected the gut microbiota and attenuated the disease (7). Our studies, along with two recent studies using additional models of murine lupus (8, 9), have established that gut microbiota is causative rather than the result of the disease. In SLE patients, one cross-sectional study showed that a greater *Bacteroidetes* to *Firmicutes* ratio was present in the fecal microbiota of SLE patients (10), whereas another cross-sectional study showed a strong association between SLE disease activity and expansion of *Ruminococcus gnavus* in the gut microbiota (11). Moreover, the fecal microbiota of SLE patients induced more Th17 differentiation than the control microbiota from healthy individuals (12). Furthermore, human studies and investigations of different mouse models have identified a couple of SLE-driving pathobionts such as *Enterococcus gallinarum* (13). It has been suggested that translocation of pathobionts due to a leaky gut leads to systemic inflammation. This is supported by evidence that the gut in mice and people with SLE is leaky (7, 11, 13–15) and evidence of bacterial translocation into extraintestinal tissues (13). Notably, *Lactobacillus* spp. are capable of reversing the leaky gut, thus attenuating lupus (7). However, it remains unclear how the mixture of 5 strains of *Lactobacillus* spp. modulated the interaction between gut microbiota and the immune system to affect disease. Specifically, whether individual *Lactobacillus* species could recapitulate the benefits of the mix had not been investigated.

In this study, we started with investigating the effects of individual *Lactobacillus* spp. in female MRL/*lpr* mice. Surprisingly, none of them was able to reproduce the benefits; instead, all 5 species acted in synergy to attenuate lupus-like disease. In addition, while the gut microbiota changes mostly did not correlate with the disease outcome, the relative abundance of two genera, *Clostridium* and *Oscillibacter*, at earlier time points was negatively correlated with lymphadenopathy. Mechanistically, we found that the mixed *Lactobacillus* spp. attenuated splenomegaly and lymphadenopathy through secreted factors and a CX_3_CR1-dependent manner. Finally, we characterized alterations of immune cell populations in lymphoid organs demonstrating that the mixed *Lactobacillus* treatment might act on multiple T cell subsets to attenuate lupus in MRL/*lpr* mice. These results suggest synergistic actions of multiple *Lactobacillus* spp. that may benefit SLE patients as a combination therapy.

## Material and Methods

### Ethics Statement

We followed the recommendations in the Guide for the Care and Use of Laboratory Animals of the NIH. The protocol was approved by the Institutional Animal Care and Use Committee (IACUC) of Virginia Tech College of Veterinary Medicine. All experiments with animals followed the guidelines provided by the IACUC protocol #18-060 and #21-003, including performing euthanasia with CO_2_.

### Mice

MRL/*lpr* (MRL/Mp-*Fas^lpr/lpr^*, stock number 00485) mice were purchased from The Jackson Laboratory (JAX). MRL/*lpr*-*Cx3cr1^gfp/gfp^* mice were generated in-house as previously described (16). Mice were bred and maintained in a specific pathogen‐free facility under the requirements of IACUC at Virginia Polytechnic Institute and State University. All *Lactobacillus* strains, *L. reuteri* (CF48-3A), *L. oris* (F0423), *L. johnsonii* (135-1-CHN), *L. gasseri* (JV-V03) and *L. rhamnosus* (LMS201), were obtained from BEI Resources. All 5 strains were freshly and separately cultured every week, then inoculated to mice as a mix or individually, twice a week at 10^9^ total CFU, from 3 weeks of age until dissection. The inoculation volume increased with age, with 100µL at 3 weeks of age, 150µL at 4 weeks of age, and 200µL for the remaining weeks. The combined culture supernatant was obtained after spinning down the bacteria and transferring the supernatant to a new tube in sterile conditions, then filtered through a 0.22µm syringe filter and stored at -80°C. The supernatant from10^9^ total CFU of bacteria was orally gavaged at 200µL at a frequency of twice a week. The same volume and frequency were used for MRS broth oral gavage. All mice were continuously monitored every week for body weight and level of proteinuria, and euthanized at 15 weeks of age. Once mouse dissection was performed, body and organ weights were recorded.

### Analysis of proteinuria, endotoxin, and antibodies

Proteinuria was determined with a Pierce Coomassie Protein Assay Kit (Thermo Scientific). Blood endotoxin was measured by using a Pierce LAL Chromogenic Endotoxin Quantitation Kit (Thermo Scientific). Anti-dsDNA IgG was measured as previously described (17). Serum total IgG concentration was determined with mouse IgG ELISA kit (Bethyl Laboratories).

### 16S rRNA sequencing analysis

Fecal samples were collected weekly directly from the anus for each mouse. Samples were stored at -80°C till being processed at the same time. Samples were homogenized, cell lysed, and DNA extracted as previously described (6, 7, 18–21). PCR were performed and purified amplicons were sequenced bidirectionally (V4 region) on an Illumina MiSeq at Argonne National Laboratory. Data analysis was performed as described previously (6, 22).

### Flow cytometry

Spleen and mesenteric lymph node (MLN) were collected and mashed in 70-µm cell strainers with C10 media (RPMI-1640 containing L-glutamine, 10% fetal bovine serum, 100 U/ml penicillin-streptomycin, 10 mM HEPES, 1 mM sodium pyruvate, 1% 100X MEM non-essential amino acids, 55 µM 2-mercaptoethanol, all from Life Technologies). For splenocytes, red blood cells were lysed with RBC lysis buffer (eBioscience). For FACS staining, cells were blocked by anti-mouse CD16/32 (eBioscience), stained with fluorochrome-conjugated antibodies, and analyzed with BD FACSAria Fusion flow cytometer (BD Biosciences). Anti-mouse antibodies used in this study include: CD3-FITC and B220-FITC (eBioscience), CD4-PerCP/Cy5.5, CD8-PE/Dazzle, PD-1-APC/Cy7, CXCR5-BV605, CD25-BV421, FOXP3-PE, CD44-APC, CD62L-APC/Cy7, CD138-PerCP/Cy5.5, CD19-BV421, CD273-PE/Dazzle, CD38-PE/Cy7 and GL7-APC (Biolegend). For intracellular staining, Foxp3/Transcription Factor Staining Kit was used to fix the cells (Biolegend). For intracellular staining of IL-10 producing cells, splenocytes or MLN cells were pre-stimulated for 4 hours with 500X Cell Stimulation Cocktail (Invitrogen) plus protein transport inhibitor (eBioscience) containing phorbol 12-myristate 13-acetate (PMA), ionomycin, brefeldin A and monensin; then stained with CD19-AF700, CD138-BV711, Foxp3-AF647 and IL-10-PE. FlowJo was used to analyze data.

### Kidney histopathology

Kidneys were fixed in formalin right after isolation. Fixed tissues were paraffin-embedded, sectioned, and stained for Periodic Acid-Schiff (PAS) at the Histopathology Laboratory at Virginia Tech College of Veterinary Medicine. Kidney histopathology score were graded by a board-certified pathologist in a blindly fashion using the following categories: glomerular lesions (cellularity, mesangial matrix, necrosis, percentage of sclerotic glomeruli, and presence of crescents, tubulointerstitial lesions and vasculitis inflammation (23).

### Analysis of tight junction transcripts

Isolated intestinal epithelial cells (IECs) were obtained as described previously (7). Reverse transcription and quantitative PCR were performed as we reported (24). Relative quantities were calculated using 18S and GAPDH as the housekeeping genes. Primer sequences for mouse *Cldn1*, *Cldn2*, *ZO1* are available upon request.

### Statistical analyses

For the comparison of two groups, unpaired student’s *t*-test was used. For the comparison of three or more groups, one-way ANOVA was used. Two-way ANOVA was used to reveal time- and group-dependent effects. Results were considered statistically significant when *p*<0.05. All analyses were performed with the GraphPad Prism software.

## Results

### Five *Lactobacillus* species act synergistically to attenuate splenomegaly and lymphadenopathy

Previously, we reported alterations of the gut microbiota in MRL/*lpr* mice that exhibited a state of dysbiosis characterized by lower abundance of the *Lactobacillaceae* bacterial family (6). This dysbiosis disrupted the balance between commensal bacteria and the immune system leading to a permanent state of inflammation. We next reported that restoring the microbiota with a mix of 5 different *Lactobacillus* species (spp.), *L. reuteri, L. oris, L. johnsonii, L. gasseri* and *L. rhamnosus*, attenuated lupus-liked clinical signs, including splenomegaly and lymphadenopathy (7). However, the underlying mechanisms were not clear. In this study, we first investigated the effects of individual species. As we showed *L. reuteri* together with an uncultured *Lactobacillus* dominated the gut microbiota of *Lactobacillus*-treated MRL/*lpr* mice (7), we hypothesized that *L. reuteri* would be the best candidate to reproduce the same beneficial effects.

**Figure 1:**
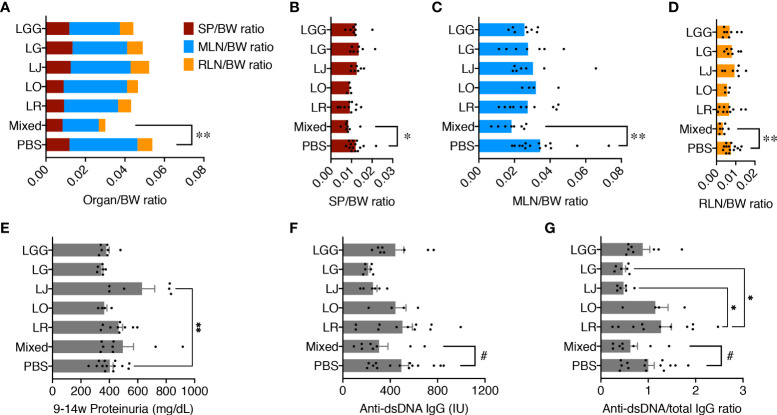
Five *Lactobacillus* species act synergistically to attenuate splenomegaly and lymphadenopathy. Female MRL/*lpr* mice were treated with indicated *Lactobacillus* spp. at 10^9^ total CFU/mouse twice a week from 3 weeks to 15 weeks of age. PBS, phosphate buffered saline control. Mixed, the mix of all 5 species. LR, LO, LJ, LG and LGG represent *L. reuteri*, *oris*, *johnsonii*, *gasseri* and *rhamnosus*, respectively. **(A)** Combined organ weight-to-body weight ratio. SP, spleen; MLN, mesenteric lymph node; RLN, renal lymph node. **(B)** SP-to-body weight ratio. **(C)** MLN-to-body weight ratio. **(D)** RLN-to-body weight ratio. **(E)** Accumulated proteinuria level from 9 weeks to 14 weeks of age. **(F)** Serum level of anti-dsDNA IgG at 15 weeks of age. IU, international units. **(G)** Serum level of anti-dsDNA IgG-to-total IgG weight ratio at 15 weeks of age. n ≥ 7 mice per group. Statistical significance (*p<0.05, **p<0.01) is shown based on one-way ANOVA. Nearly significant differences between PBS and Mixed are shown as ^#^ *p*<0.1.

Female MRL/*lpr* female mice were orally gavaged, twice a week at 10^9^ CFU/mouse, freshly cultured *Lactobacillus* spp. either individually or as a mix from 3 weeks of age (pre-disease) till 15 weeks of age (late disease). Upon euthanasia, the weight of different organs including spleen, mesenteric lymph node (MLN), and renal lymph node (RLN) was measured, and the organ-to-body weight ratios were calculated. There were no differences except for the mix, which significantly reduced the combined weight of all 3 lymphoid organs compared to the PBS control (**Figure 1AM**), suggesting that only the mix was beneficial against splenomegaly and lymphadenopathy. When looking at the lymphoid organs individually, we observed similar results with significantly smaller organs for the mix (**Figures 1B–D**). Each strain individually did not have enough impact to exert a beneficial effect; instead, the 5 species worked synergistically to attenuate splenomegaly and lymphadenopathy.

We previously reported that lupus nephritis was linked to gut microbiota dysbiosis (7). We thus tested the levels of proteinuria, but no differences were found except for *L. johnsonii*, which significantly exacerbated glomerulonephritis over the PBS control (**Figure 1E**). Notably, the MRL/*lpr* mice in our in-house colony, similar to those housed at The Jackson Laboratory, have lost the kidney disease phenotype (25), leading to low levels of proteinuria throughout the experiment. As expected, kidney histopathological analysis revealed no difference in glomerular and tubulointerstitial lesions as well as leukocyte infiltration (**Supplementary Figure 1**). However, the serum level of anti-dsDNA IgG, a typical pathogenic autoantibody in lupus, as well as the anti-dsDNA IgG to total IgG ratio in the blood, were moderately decreased by the mix, although *L. johnsonii* and *L. gasseri* individually had similar effects (**Figures 1F, G**).

These results indicate that none of the species individually recapitulated the benefits of the mix. Instead, these *Lactobacillus* spp. acted synergistically to attenuate splenomegaly and lymphadenopathy.

### Gut microbiota during early disease is associated with the decrease of gut-draining lymph node size

**Figure 2:**
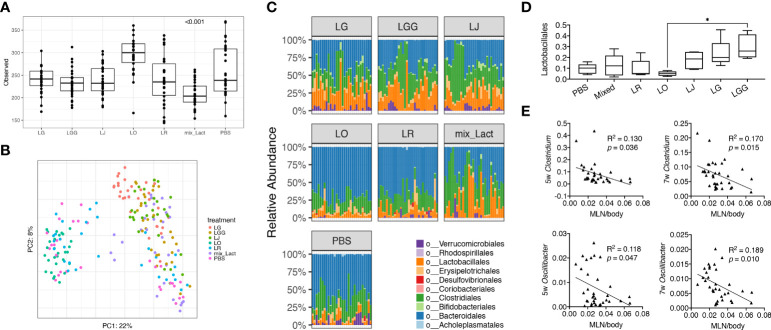
Gut microbiota at early stage is associated with the decrease of gut-draining lymph node size. Fecal microbiota samples were collected at 3, 5, 7, 9, 11 and 13 weeks of age (n = 5 mice per group) and subjected to 16S rRNA sequencing. **(A)** Observed OTUs (*p*<0.001). **(B)** Principal coordinate analysis of fecal microbiota composition. **(C)** Time-dependent changes of fecal microbiota at the order level. **(D)** The relative abundance of the order *Lactobacillales*. Statistical significance (*p<0.05) is shown based on one-way ANOVA. **(E)** Correlation analysis of the relative abundance of the genera *Clostridium* and *Oscillibacter* at 5 and 7 weeks of age against the MLN-to-body weight ratio at 15 weeks of age.

To study the associations between changes of disease phenotype and the composition of gut microbiota, we analyzed the fecal samples collected from mice treated with each *Lactobacillus* individually or in combination by using 16S rRNA sequencing. Looking at the observed operational taxonomic units (OTUs), we found that the gut microbiota from mice treated with the mix was significantly less diverse than that treated with the PBS control (**Figure 2A**), likely due to the enrichment of *Lactobacillus* spp. In addition, we performed principal coordinate analysis and found the gut microbiotas from PBS, *L. reuteri* and *L. oris* treated groups were clustered together, whereas the gut microbiotas from the mix, *L. johnsonii, L. gasseri* and *L. rhamnosus* treated groups were clustered together (**Figure 2B**). Analysis at the order level confirmed these observations (**Figure 2C**). The order of *Lactobacillales*, in particular, followed this pattern with the *L. rhamnosus* treated group harboring significantly more *Lactobacillales* than the *L. oris* group (**Figure 2D**). These results did not correlate with the observed disease phenotype, where only the mix was beneficial. However, it was evident that the relative abundance of genera *Clostridium* and *Oscillibacter* at earlier time points of 5 and 7 weeks of age was negatively correlated with the size of MLNs at 15 weeks of age (**Figure 2E**; **Supplementary Table 1**). No correlation was observed between the size of endpoint MLNs and the relative abundance of these genera at 13 weeks of age (**Supplementary Figure 2A**), suggesting that the changes of gut microbiota may precede the change of disease.

### *Lactobacillus* spp. attenuate splenomegaly and renal lymphadenopathy through secreted factors and a CX_3_CR1-dependent mechanism

**Figure 3:**
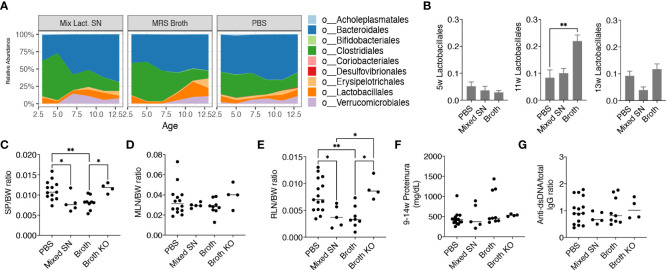
*Lactobacillus* spp. attenuate splenomegaly and renal lymphadenopathy through secreted factors and a CX_3_CR1-dependent mechanism. Female MRL/*lpr* mice were orally gavaged with PBS, the combined culture supernatant of the 5 *Lactobacillus* spp. (Mixed SN), or MRS broth (Broth) twice a week at 200 μL/mouse from 3 weeks to 15 weeks of age. In some experiments, female MRL/*lpr-Cx3cr1^gfp/gfp^* mice were treated with MRS broth (Broth KO) following the same protocol. **(A)** Time-dependent changes of fecal microbiota (n = 5 mice per group). **(B)** The relative abundance of the order *Lactobacillales* at 5, 11 and 13 weeks of age. **(C)** SP-to-body weight ratio. **(D)** MLN-to-body weight ratio. **(E)** RLN-to-body weight ratio. **(F)** Accumulated level of proteinuria from 9 to 14 weeks of age. **(G)** Anti-dsDNA-to-total IgG ratio. n ≥ 7 mice per group. Statistical significance (*p<0.05, **p<0.01) is shown based on one-way ANOVA.

After uncovering that only the mix exerted beneficial effects, we further investigated whether it was the bacteria themselves or their secretion that improved the disease outcome. We thus treated female MRL/*lpr* mice with the combined culture supernatant of all 5 *Lactobacillus* spp. using the optimal culture medium of *Lactobacillus*, MRS broth, as the negative control. Interestingly, MRS broth transiently but significantly expanded endogenous *Lactobacillales* when mice were 11 weeks of age, whereas the gut microbiota following supernatant treatment was similar to that of the PBS control (**Figures 3A, B**), even though the bacterial diversity of the gut microbiota was significantly lower upon treatment with the culture supernatant (**Supplementary Figure 2B**). Nonetheless, the culture supernatant was effective in reducing splenomegaly and renal lymphadenopathy, although no differences were observed for the size of MLN, proteinuria and anti-dsDNA IgG to total IgG ratio (**Figures 3C-G**). The latter was consistent with no differences in the endotoxin level (**Supplementary Figure 2C**) and tight junction gene expression (**Supplementary Figure 2D**). The MRS broth, presumably through enriching endogenous *Lactobacillus* spp., exerted similar benefits on splenomegaly and renal lymphadenopathy (**Figures 3C, E**).

Furthermore, we were interested in CX_3_CR1, a receptor that helps to present commensal bacterial antigens to the proximal lymph nodes, limiting the immune response against beneficial bacteria while removing harmful bacteria (26). We previously generated MRL/*lpr*-*Cx3cr1^gfp/gfp^* mice where *GFP* replaced the coding region of the *Cx3cr1* gene leading to global knockout of CX_3_CR1 (16). By gavaging these mice with the MRS broth, we found that the benefits of the broth were eliminated in the knockout mice (**Figures 3C, E**), suggesting a CX_3_CR1-dependent mechanism by which *Lactobacillus* spp. improve splenomegaly and renal lymphadenopathy.

Together, these results suggest that *Lactobacillus* spp. attenuate splenomegaly and renal lymphadenopathy through secreted factors and a CX_3_CR1-dependent mechanism.

### *Lactobacillus* spp. control inflammation through regulating splenic and MLN immune cell populations

After observing the beneficial effects of mixed *Lactobacillus* spp., their culture supernatant, and MRS Broth, we sought to understand the immunological mechanism by which these treatments inhibited inflammation towards to a more balanced environment leading to decreased lymphoid organ sizes. We first analyzed the germinal center (GC) reaction in the spleen and MLN. Gating on CD4+PD−1+CXCR5+ follicular T helper (Tfh) cells (**Supplementary Figure 3A**) and CD19+GL7+CD38−/^low^ GC-B cells (**Supplementary Figure 3B**), we found mostly no difference in these populations (**Supplementary Figures 3C, D**), although the mixed *Lactobacillus* spp. significantly induced more GC-B cells over the PBS control in the MLN (**Supplementary Figure 3D**). We also gated on the recently identified CD273+ regulatory B (Breg) cells (27–29) (**Supplementary Figure 3E**), and the mixed *Lactobacillus* spp. was able to significantly induce more of these Breg cells in the MLN (**Supplementary Figure 3F**). It is possible that the antibody-producing GC-B cells and immunosuppressive Breg cells counter their respective effects; therefore, overall, we did not observe significant alterations of spleen or MLN GC reaction by the treatment groups.

**Figure 4:**
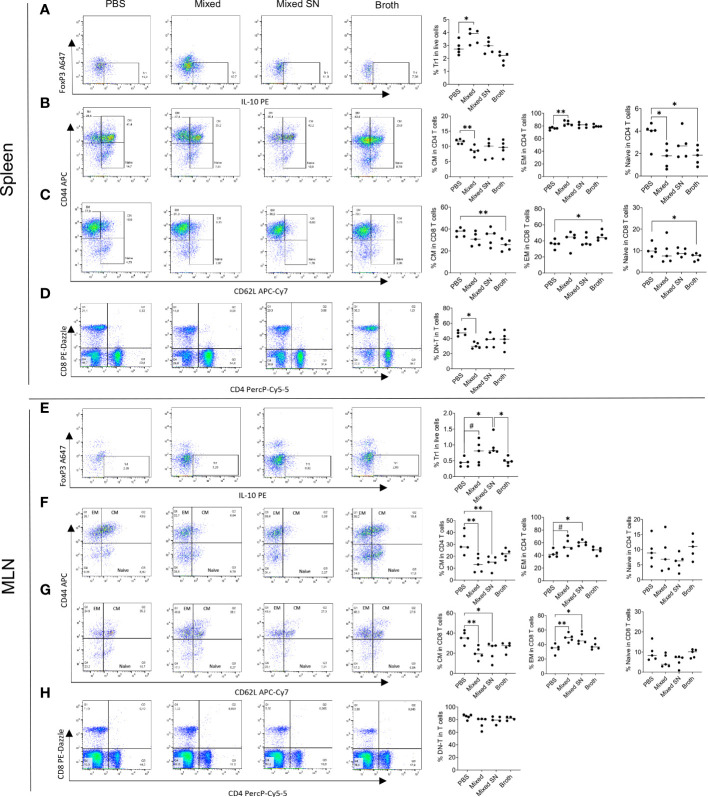
*Lactobacillus* spp. control inflammation through regulating splenic and MLN immune cell populations. **(A–D)** Flow cytometry analysis of splenocytes. **(E–H)** Flow cytometry analysis of MLN cells. **(A–E)** Frequency of IL-10-expressing type 1 regulatory T (Tr1) cells. Plots were pre-gated on CD4^+^ T cells. **(B, F)** Frequencies of central memory (CM) and effector memory (EM) T cells as well as naïve T cells within CD4^+^ T cells. Plots were pre-gated on CD4^+^ T cells. **(C–G)** Frequencies of CM and EM T cells as well as naïve T cells within CD8^+^ T cells. Plots were pre-gated on CD8^+^ T cells. **(D–H)** Frequency of double-negative T (DNT) cells. Plots were pre-gated on T cells. Statistical significance (*p<0.05, **p<0.01) is shown based on one-way ANOVA. Nearly significant differences are shown as ^#^ *p*<0.1.

Next, we analyzed several populations of IL-10 producing immunoregulatory cells. These included CD4^+^Foxp3^−^IL-10^+^ type 1 regulatory T (Tr1) cells (**Figure 4A**), CD4^+^Foxp3^+^IL-10^+^ regulatory T (Treg) cells, CD19^+^IL-10^+^ Breg cells, and CD138^+^IL-10^+^ plasma cell (PC)-derived Breg cells (**Supplementary Figure 4A**). We found consistent upregulation of Tr1 cells in the spleen and MLN with the mixed *Lactobacillus* treatment (**Figures 4A–E**). This observation aligns well with our published results on Tr1 cells as IL-10 producers in MRL/*lpr* mice (7). Interestingly, the combined culture supernatant of all 5 *Lactobacillus* spp. was also able to significantly increase the percentage of Tr1 cells in the MLN (**Figure 4E**). In addition, we observed decreased percentages of FOXP3+IL-10^+^ Treg cells and CD138+ PC-Breg cells in the MLN with both the mixed *Lactobacillus* and MRS broth treatments (**Supplementary Figure 4B**). These results suggest that Tr1 cells may be responsible for the immunoregulatory effects of the mixed *Lactobacillus* treatment. Notably, while CD4^+^Foxp3^+^IL-10^+^ Treg cells were reduced in the MLN (**Supplementary Figure 4B**), CD25^+^FOXP3^+^ Treg cells were significantly increased in the MLN with the mixed *Lactobacillus* treatment (**Supplementary Figure 4C**), suggesting that Treg cells may produce cytokines other than IL-10, such as TGFβ, to suppress inflammation.

Moreover, oral gavage of *Lactobacillus* spp. decreased the percentage of central memory T (T_CM_) cells while increasing that of effector memory T (T_EM_) cells in both CD4^+^ and CD8^+^ compartments in the spleen (**Figures 4B, C**) and MLN (**Figures 4F, G**). Since T_CM_ cells are known to sustain persistent inflammation (30), the conversion of them into short-lived T_EM_ cells may be beneficial. Notably, the MRS broth exerted similar benefits within the CD8^+^ compartment in the spleen (**Figure 4C**), whereas the combined culture supernatant regulated T_CM_ and T_EM_ cells in a similar fashion as mixed *Lactobacillus* spp. in the MLN (**Figures 4F, G**). Naïve T cells, on the other hand, were suppressed by mixed *Lactobacillus* spp. or MRS broth in the spleen (**Figures 4B–C**). These results suggest that *Lactobacillus* spp. may target memory T cells to suppress inflammation in MRL/*lpr* mice.

Furthermore, the mixed *Lactobacillus* treatment significantly downregulated the percentage of double-negative T (DNT) cells in the spleen (**Figure 4D**), which play a proinflammatory role by secreting IL-17 that aggravates the autoimmune environment, particularly in lupus patients (31–34). The percentage of DNT cells in the MLN, on the other hand, did not change (**Figure 4H**).

Together, these results suggest that *Lactobacillus* spp. might act on T cells to attenuate splenomegaly and lymphadenopathy.

## Discussion

In this study, we showed that restoring the microbiota with a mix of 5 different *Lactobacillus* spp., *L. reuteri, L. oris, L. johnsonii, L. gasseri* and *L. rhamnosus*, was able ameliorate lupus-like clinical signs, reducing splenomegaly and lymphadenopathy. However, none of the strains individually could replicate the same positive effects that the mix *Lactobacillus* spp. showed, suggesting a potential cooperation among the species that helps to multiply each individual strength. In addition, the combined culture supernatant of *Lactobacillus* spp. attenuated splenomegaly and renal lymphadenopathy suggesting that the bacteria benefit by releasing certain secreted factors. Interestingly, oral administration of MRS broth exerted the same benefits probably by creating a favorable environment that increased the relative abundance of endogenous *Lactobacilliales*. Moreover, *Cx3cr1* deficiency in MRL/*lpr* mice abrogated the benefits showing a comparable level of disease as the control group, suggesting that the MRS broth, and thus the endogenous *Lactobacillus* spp., may exert protective effects through a CX_3_CR1-dependent mechanism. The mechanistic pathway of the mix of *Lactobacillus* spp. increasing the percentages of FOXP3-negative Tr1 cells in both spleen and MLN suggests a possible probiotic-mediated control of inflammation that may happen in both MRL/*lpr* mice and lupus patients. Furthermore, oral gavage of *Lactobacillus* spp. increased the relative abundance of T_EM_ cells in the lymphoid organs while decreasing T_CM_ cells. Finally, a lower percentage of DN T cells, which plays a detrimental role in SLE, was observed in the spleen, suggesting an additional protective mechanism provided by the mix of *Lactobacillus* spp.

Our novelty resides in the fact that individual *Lactobacillus* strains could not replicate the effects of the mix. This result was surprising to us and was not reported previously. In addition, while we did report the effect of the mixed *Lactobacillus* spp. on Tr1 cells, the new information we are presenting is the effects of the mixed culture supernatant and MRS broth. The mixed *Lactobacillus* group was used as the positive control. We advance the previous findings by explaining how *Lactobacillus* spp. could potentially exert the beneficial effects. Here we show data suggesting that they do so through secreted factors produced by the administered probiotics. We also show similar benefits due to MRS broth-mediated enrichment of local *Lactobacillus* spp. found in the gut microbiota.

MRL/*lpr* mice have been extensively used as a murine model of lupus. These mice spontaneously develop autoimmune disease resembling SLE patients due to multiple SLE susceptibility loci present in the MRL background as well as a spontaneous mutation in the *Fas* gene (35). Fas is a type I membrane protein of the tumor necrosis factor receptor (TNFR) superfamily that is expressed on activated lymphocytes and in the thymus which induces lymphocyte apoptosis upon ligation by the Fas ligand. The *Fas* defect triggers the survival of self-reactive lymphocytes common in autoimmunity (36). Therefore, the *Fas* mutation accelerates the disease in MRL mice leading lymphoproliferation with progressive organ failures. Many features of the MRL/*lpr* mouse, including autoantibodies, glomerulonephritis, and infiltrates of proinflammatory B and T cells in peripheral tissues such as the kidney, resemble clinical symptoms in SLE patients. In addition, like human SLE, the MRL/*lpr* mouse develops progressive nonmalignant lymphoproliferation that contributes to an early mortality (37).

We recently published a study where disease manifestations in MRL/*lpr* mice can differ among animal facilities, suggesting a role for environment factors (25). Notably, the gene loci responsible for splenomegaly and lymphadenopathy, which are chromosomes 4, 5 and 7, are not the same as that for glomerulonephritis, which is chromosome 10. We found that comparing our in-house mice with those obtained from The Jackson Laboratory, there were differences in lymphoproliferation (splenomegaly and lymphadenopathy), but the loss of kidney disease (glomerulonephritis) was a shared phenomenon. Due to this significant phenotypic drift, we decided to focus on the splenomegaly and lymphadenopathy in the current study.

To further investigate the mechanism by which the gut microbiota modulates lupus disease progression, we characterized the gut microbiota and established correlations between gut bacteria and disease phenotype. Although less diversity traditionally means unhealthier gut microbiota (38), in the context of autoimmune disease, *Lactobacillus* spp. induces a positive change in the ecosystem, supporting microbiota stability through their metabolic activities, interacting with the host to provide a low inflammatory state or to revert a proinflammatory state, and resisting the colonization from pathogens. In contrast, higher microbiota diversity in this setting seems to be associated with disease or dysfunction, leading to competition instead of cooperation, which undermines the stability of the microbial community. *Lactobacillus* spp., on the other hand, corrects this. Notably, the introduction of probiotics such as *Lactobacillus* spp. can be a starting point to redirect the gut microbiota towards to a better and healthier profile. It has been reported that major interventions in gut microbiota can temporarily reduce diversity, as the process of enriching certain bacteria could lead to a change in composition and, through competitive interactions, reduce diversity (39).

Interestingly, the relative abundance of genera *Clostridium* and *Oscillibacter* was negatively correlated to the size of MLN. *Clostridium* is a genus with butyrate-producing gram-positive bacteria (40). Known to exert beneficial effects for intestinal homeostasis, *Clostridium* spp. can have an important role in attenuating inflammation due to their cellular components and metabolites, likely through energizing intestinal epithelial cells, stimulating intestinal barrier health, and educating the immune system. Moreover, a member of the *Oscillibacter* genus, *O. valericigenes*, is a valerate- and butyrate-producing anaerobic gram-negative non-sporulating bacterium (41). *O. valericigenes* has been found in the human gut microbiota. In particular, *O. valericigenes* was found more abundant in healthy individuals compared to patients diagnosed with Crohn’s disease (42). An important short-chain fatty acid (SCFA), butyrate has been shown to successfully prevent inflammation through Treg induction (43) and down-regulating the expression of pro-inflammatory cytokines (44). Therefore, it is conceivable, while yet to be elucidated, that the negative correlation between mesenteric lymphadenopathy and the genera *Clostridium* and *Oscillibacter* may be due to the ability of the bacteria to produce the anti-inflammatory butyrate. It is likely that the presence or products of *Lactobacillus* spp. facilitated the proliferation and/or survival of these two genera, even though we do not know the mechanisms at this time. It is known, however, that *Lactobacillus* spp. are SCFA producers and can be butyrate-producing bacteria that alleviate intestinal permeability and maintain barrier function. Butyrate can not only inhibit pathogenic bacteria but also stimulate the growth of beneficial bacteria (45), a notion that could be associated with the enhancement of the relative abundance of *Oscillibacter* and *Clostridium*.

The secreted factors in the culture supernatant, potentially bacterial metabolites, showed the same benefits as the mixed *Lactobacilli* themselves. Bacterial metabolites are important particularly for food digestion and metabolic pathways of dietary carbohydrates, protein, fat and vitamins (46). SCFAs such as acetate, propionate and butyrate are bacterial metabolites generated from the fermentation of dietary fiber by the gut microbiota. They play immune modulatory roles and are involved in the maintenance of colonic mucosal health by stimulating the mucous layer, producing antimicrobial peptides, and serving as anti-inflammatory agents due to their regulatory effects on gene expression (47). Besides SCFAs, *Lactobacillus* spp. are known for their production of antimicrobial compounds, including biosurfactants, lactic acid, hydrogen peroxide, bacteriocins and bacteriocin-like peptides that could inhibit pathogen growth (48). It is likely that the metabolites of *Lactobacillus* spp., such as indole molecules, bind to the aryl hydrocarbon receptor (AhR) of T cells leading to Tr1 differentiation (49–52). Further research is necessary to identify specific secreted factors that recapitulate the benefits of *Lactobacillus* spp. on lupus.

CX_3_CR1 is important for maintaining intestinal homeostasis and controlling pathogen challenges (26, 53). CX_3_CR1, or the fractalkine receptor, in conjunction with its ligand CX_3_CL1, mediates the translocation of the antigen-presenting cells from the blood to the gut, MLN, kidney and brain. The role of CX_3_CR1-expressing cells in the intestinal epithelium is to capture bacteria and antigens present in the gut lumen and to transport them to the MLN, where T cells are activated (54). Interestingly, however, *Cx3cr1*-deficient mice display markedly increased translocation of commensal bacteria into the MLN leading to gut inflammation (55), suggesting that CX_3_CR1-expressing cells also have a regulatory function and promote homeostasis between commensal bacteria and immune system. Our study supports the important role of CX_3_CR1 as an antigen carrier and an immune regulator, where the lack of the receptor abrogates the protective effects of *Lactobacillus* spp. enriched by the MRS broth leading to aggravated lupus.

Type 1 regulatory T (Tr1) can prevent and downregulate undesired immune responses to pathogenic and non-pathogenic antigens (such as autoantigens) and are associated with long-term tolerance. Tr1 cells produce IL-10 as well as TGF-β, but they can also produce variable amounts of other cytokines depending on the microenvironment and disease context (56). Notably, the ability to produce IL-10 does not equal to suppressor activity (57); however, IL-10-producing Tr1 cells do have suppressor functions that are independent from FOXP3 (58). Our results suggest that *Lactobacillus* treatment can promote an anti-inflammatory environment in the gut of lupus-prone mice through increasing the frequency of Tr1 cells. T_CM_ and T_EM_ cells are antigen-specific memory T cells to viruses or other microbial molecules and their primary function is to trigger an immune response upon reintroduction of relevant pathogen into the body (59). T_CM_ cells can sometimes react to novel antigens, potentially caused by intrinsic diversity and breadth of the T cell receptor (60). These memory T cells could cross-react to environmental or resident antigens in our body (such as commensal bacteria in the gut) and proliferate. The cross-reactivity mechanism may be important to help maintain the T_CM_ population at mucosal surfaces. However, through the same mechanism, T_CM_ cells can sustain autoimmune response by proliferating in the secondary lymphoid organs and producing a persistent inflammatory state (61). T_EM_ cells, in comparison, present an immediate, but not sustained, defense at the pathogen’s sites of entry. *Lactobacillus* treatment promotes a T_EM_ response while suppressing T_CM_ cells, thereby present an immediate response with subsequent resolution instead of prolonging the autoimmune response. Notably, the frequencies of T_CM_ cells decreased in the spleen and MLN that decreased their weights. This suggests that decreases of T_CM_ cells may be more critical changes induced by *Lactobacillus* spp. than relatively increased frequencies of Tr1 cells, as the latter increased while the organ weight decreased, leading to no change in actual cell numbers. DNT cells are characteristically elevated in several autoimmune diseases including SLE, and expanded DNT cells in inflamed tissues contribute to disease pathogenesis (34). DNT cells can produce inflammatory cytokines IL-17 and IFN-γ, which contribute to the pathogenesis of kidney damage in patients with SLE (62). Therefore, the suppression of DNT cells may be another reason that lupus-like clinical signs are improved by *Lactobacillus* treatment. Altogether, our results suggest the probiotic bacteria may target T cells to control the autoimmune response in MRL/*lpr* mice.

In conclusion, this study advances our understanding of the mechanisms of how changes of the gut microbiota regulate SLE-associated immune responses in mice. Future studies will determine if these results can be replicated in human SLE patients. The ultimate goal is to uncover novel, gut microbiota-related pathogenic pathways of SLE that enable the identification of new therapeutic targets, for which the modification of gut microbiota—through diet modulation and/or probiotic supplementation—can be a cost-effective approach to complement the current SLE treatment strategies.

## Data Availability Statement

The datasets presented in this study can be found in online repositories. The names of the repository/repositories and accession number(s) can be found below: https://www.ncbi.nlm.nih.gov/, PRJNA827847.

## Ethics Statement

The animal study was reviewed and approved by The Institutional Animal Care and Use Committee (IACUC) of Virginia Tech College of Veterinary Medicine (Animal Welfare Assurance Number: A3208-01).

## Author Contributions

XL designed the study. XC-P, QM, RL, BS, LA, JZ, MP and SC performed the experiments. XC-P, SS, CR, SA and XL analysed the data. TC scored the pathology slides. XC-P and XL wrote the manuscript. All authors contributed to the article and approved the submitted version.

## Funding

This work was supported by internal funding from VMCVM and NIH grants AR067418 and AR073240 (Luo).

## Acknowledgments

We thank to Sarah Owens and Husen Zhang for assistance on Illumina MiSeq sequencing and bioinformatics analysis. In addition, we thank Melissa Makris for flow cytometry analysis.

## Conflict of Interest

The authors declare that the research was conducted in the absence of any commercial or financial relationships that could be construed as a potential conflict of interest.

## Publisher’s Note

All claims expressed in this article are solely those of the authors and do not necessarily represent those of their affiliated organizations, or those of the publisher, the editors and the reviewers. Any product that may be evaluated in this article, or claim that may be made by its manufacturer, is not guaranteed or endorsed by the publisher.
