# Supplementary material for: Lactobacillus spp. act in synergy to attenuate splenomegaly and lymphadenopathy in lupus-prone MRL/lpr mice
Source: Front Immunol. 2022 Jul 28;13:923754. doi: 10.3389/fimmu.2022.923754 (PMC9368192; doi:10.3389/fimmu.2022.923754)
Supplement: Supplementary file 1 [file DataSheet_1.docx]

**Supplemental Information**

*Lactobacillus* spp. act in synergy to attenuate splenomegaly and lymphadenopathy in lupus-prone MRL/*lpr* mice

Cabana-Puig *et al*.

Figure S1-S4

Table S1 (a separate file)


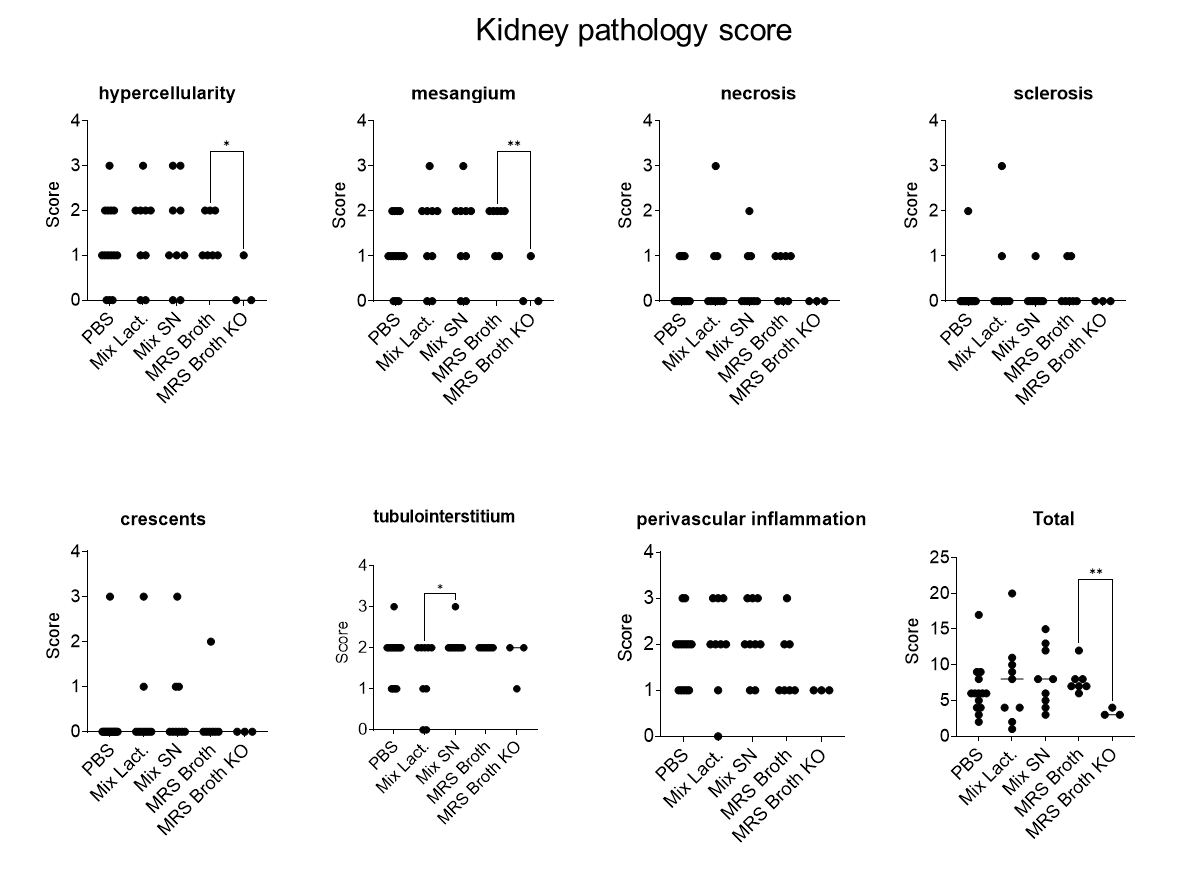


**Figure S1.** kidney histopathological scores for female mice are shown. Total score is the sum of all the other scores. n = 3, 7 or 8 mice per group; **p* < 0.05, ***p* < 0.01.

**Figure S2**. Fecal microbiota samples were collected at 3, 5, 7, 9, 11 and 13 weeks of age (n = 5 mice per group) and subjected to 16S rRNA sequencing. (**A**) Lack of correlation between the relative abundance of two genera at 13 weeks of age and the size of endpoint MLNs (*p*>0.05). (**B**) Alpha-diversity shown as observed OTUs (*p* <0.001). (**B**) Level of endotoxin in the serum. (**C**) Transcript levels of tight junction proteins in intestinal epithelial cells of 15-week-old mice. n = 5 mice per group; ^#^*p* < 0.1, **p* < 0.05.


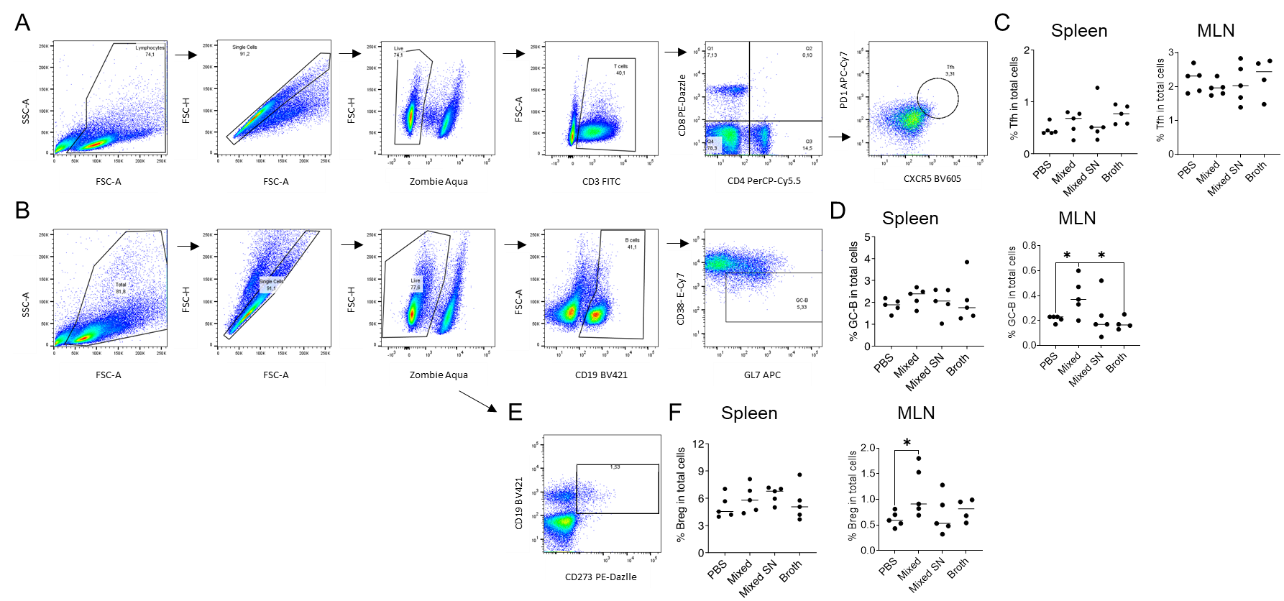


**Figure S3.** (**A**) Gating strategy for PD1^+^CXCR5^+^ T follicular helper cells (Tfh) as determined by flow cytometry. (**B**) Gating strategy for CD38^+^GL7^+^ germinal center B cells (GC-B). (**C**) Frequencies of Tfh in the spleen and mesenteric lymph node (MLN). (**D**) Frequencies of GC-B in the spleen and MLN. (**E**) Gating strategy for CD19^+^CD273^+^ B regulatory cells (Breg). (**F**) Frequencies of Bregs in the spleen and MLN. n = 5 mice per group; **p* < 0.05.


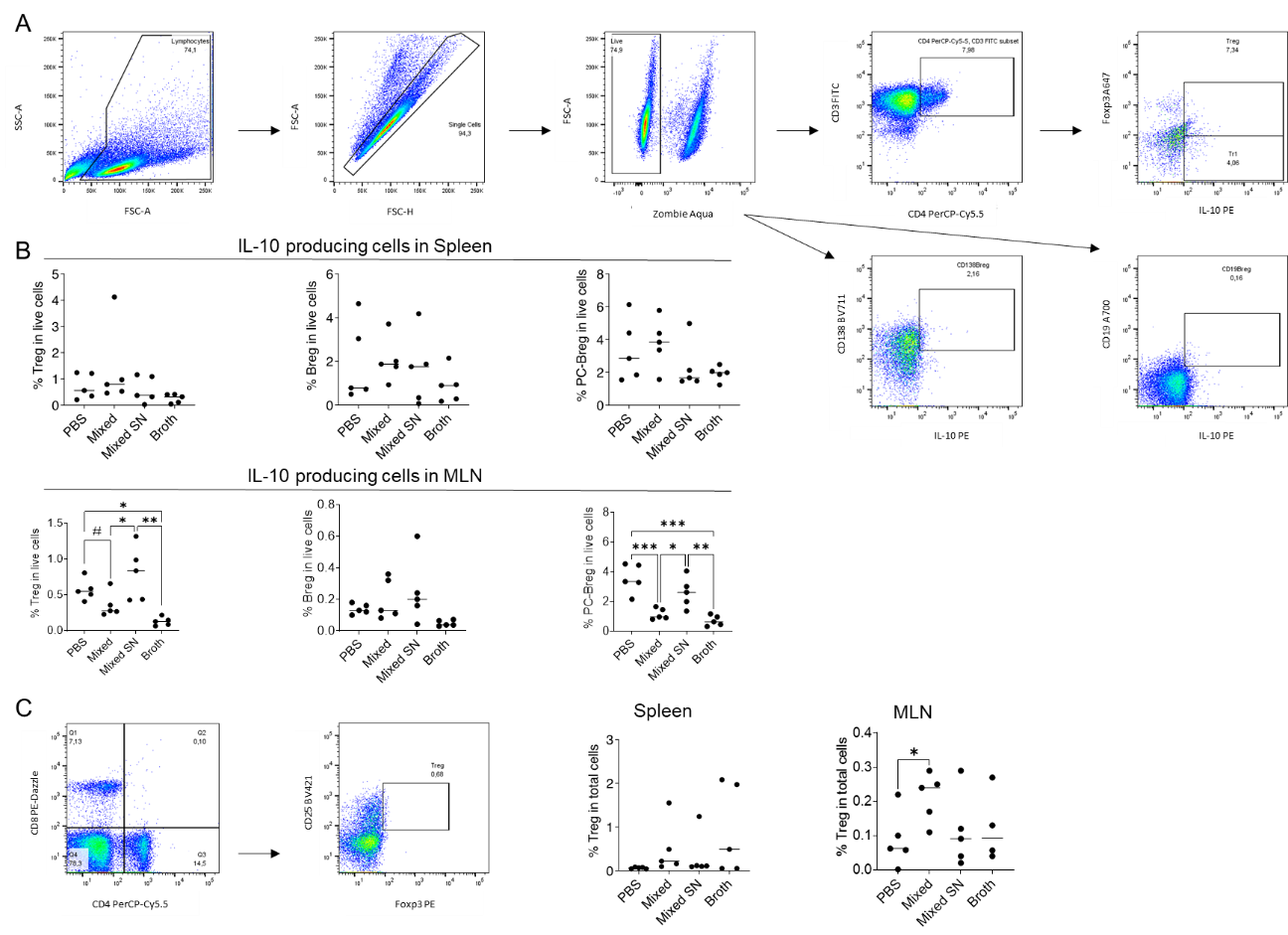


**Figure S4.** (**A**) Gating strategy for Foxp3^+^IL-10^+^ cells T regulatory cells (Treg), CD138^+^IL-10^+^ B regulatory plasma cells (PC-Breg) and CD19^+^IL-10^+^ Breg as determined by flow cytometry. (**B**) Frequency of each cell population in the spleen and MLN. (**C**) Gating strategy for CD25^+^Foxp3^+^ Treg and frequencies of such Tregs in the spleen and MLN. n = 5 mice per group; **p* < 0.05.
